# A comparative analysis of drinking water employing metagenomics

**DOI:** 10.1371/journal.pone.0231210

**Published:** 2020-04-09

**Authors:** Kyle D. Brumfield, Nur A. Hasan, Menu B. Leddy, Joseph A. Cotruvo, Shah M. Rashed, Rita R. Colwell, Anwar Huq

**Affiliations:** 1 Maryland Pathogen Research Institute, University of Maryland, MD, College Park, United States of America; 2 University of Maryland Institute for Advanced Computer Studies, University of Maryland, College Park, MD, United States of America; 3 CosmosID Inc., Rockville, MD, United States of America; 4 Essential Environmental and Engineering Systems, Huntington Beach, CA, United States of America; 5 Joseph Cotruvo and Associates LLC, Washington, DC, United States of America; University of Minnesota Twin Cities, UNITED STATES

## Abstract

The microbiological content of drinking water traditionally is determined by employing culture-dependent methods that are unable to detect all microorganisms, especially those that are not culturable. High-throughput sequencing now makes it possible to determine the microbiome of drinking water. Thus, the natural microbiota of water and water distribution systems can now be determined more accurately and analyzed in significantly greater detail, providing comprehensive understanding of the microbial community of drinking water applicable to public health. In this study, shotgun metagenomic analysis was performed to determine the microbiological content of drinking water and to provide a preliminary assessment of tap, drinking fountain, sparkling natural mineral, and non-mineral bottled water. Predominant bacterial species detected were members of the phyla *Actinobacteria* and *Proteobacteria*, notably the genera *Alishewanella*, *Salmonella*, and *Propionibacterium* in non-carbonated non-mineral bottled water, *Methyloversatilis* and *Methylibium* in sparkling natural mineral water, and *Mycobacterium* and *Afipia* in tap and drinking fountain water. Fecal indicator bacteria, i.e., *Escherichia coli* or *enterococci*, were not detected in any samples examined in this study. Bacteriophages and DNA encoding a few virulence-associated factors were detected but determined to be present only at low abundance. Antibiotic resistance markers were detected only at abundance values below our threshold of confidence. DNA of opportunistic plant and animal pathogens was identified in some samples and these included bacteria (*Mycobacterium* spp.), protozoa (*Acanthamoeba mauritaniensis* and *Acanthamoeba palestinensis*), and fungi (*Melampsora pinitorqua* and *Chryosporium queenslandicum*). Archaeal DNA (*Candidatus Nitrosoarchaeum*) was detected only in sparkling natural mineral water. This preliminary study reports the complete microbiome (bacteria, viruses, fungi, and protists) of selected types of drinking water employing whole-genome high-throughput sequencing and bioinformatics. Investigation into activity and function of the organisms detected is in progress.

## Introduction

Access to safe drinking water (DW) is considered a fundamental human right, yet it is estimated that globally more than two billion people suffer from a lack of safely managed DW services [1]. During the late nineteenth and early twentieth centuries, major cities in the U.S. adopted filtration and disinfection water treatment methods, significantly reducing mortality rates and incidence of disease associated with contaminated water [2]. Thereafter, waterborne disease outbreaks associated with conventional source water declined. Unfortunately, legionellosis, caused by inhalation of *Legionella* spp. contaminated aerosols from water distribution and plumbing, remains a concern since this disease accounts for roughly 60 percent of reported waterborne disease outbreaks in the U.S. and has emerged recently as a leading cause of reported deaths associated with contaminated water [3,4].

Under the Safe Drinking Water Act of 1974, the United States Environmental Protection Agency (USEPA) regulates public DW supplies. The United States Food and Drug Administration (USFDA), uses USEPA standards as the basis for regulating bottled water (BW) in interstate commerce. State enforcement of public DW standards protect against both naturally occurring and man-made contaminants in water entering a drinking water distribution system (DWDS) from municipal treatment facilities. The U.S. national drinking water Maximum Contaminant Level, under the Revised Total Coliform Rule [5], is less than one fecal coliform per 100 mL of water, in addition to filtration and disinfection requirements that depend upon the source. Certain bacterial and fungal species present in natural source water promote biodegradation of organic and inorganic matter, which can enhance biological stability and lower concentrations of micropollutants [6,7]. Other microorganisms pose potential health concerns. Municipal water treatment facilities eliminate or at least significantly reduce the number of pathogenic microorganisms in finished DW. Thus, municipal water is not expected to be sterile but must be microbially safe.

To limit microbial regrowth in finished DW, disinfectants, e.g., additional free chlorine or monochloramine, are added to the water prior to distribution, therefore residuals should be present in DW if they have not dissipated in transit [8,9]. However, such disinfectant residuals can introduce selective pressure, that may result in communities of disinfectant-resistant microorganisms [10–13]. For example, chlorination has been shown to greatly affect microbial community structure in DWDS [14]. Ridgway and Olson showed a possible selection for chlorine-tolerant microorganisms in chlorinated water as bacteria isolated from a chlorinated DWDS were more resistant to both combined and free forms of chlorine compared to bacteria isolated from an unchlorinated DWDS [13]. Differential resistance to monochloramine in bacterial populations has also been observed in certain genera detected in DWDS, including *Legionella*, *Escherichia*, and *Sphingomonas*, and *Mycobacterium* [12].

Furthermore, regrowth or after growth of microorganisms in treated DW, including BW, can occur [10,15–18]. Uncontrolled growth of bacteria, notably biofilm bacteria, in water mains and premise plumbing during delivery is well documented and can introduce operational issues within distribution systems, resulting in deterioration of color and taste or causing potential human hygiene problems [8,10,17,19,20]. Complex interactions also can develop between microorganisms and their environment and lead to metabolism of biologically available nutrients, particle deposition and sediment re-suspension, appearance of potential inhibitory substances, and biofilm formation. Microbial response to environmental conditions, notably temperature, also can contribute to changes in microbial water quality during distribution [8].

Thus, a major challenge is being able to measure the total microbial content of water accurately [21]. Traditionally, it has been assumed that indicator microorganisms provide adequate assurance for the microbial safety of water. Culture-dependent methods are used to detect and enumerate indicator organisms and have been remarkably successful in improving DW quality and safety, but do not detect all microorganisms present in that water. Metagenomic analysis employing high-throughput sequencing coupled with bioinformatics has gained attention during the past decade, allowing detection, identification, and characterization of all microorganisms present in DWDS [22–24]. Inferences of infectious potential of detected microbial species is determined by detecting genes coding for pathogenic and metabolic properties [24,25]. Thus, bacteria, viruses, fungi, and protists now can be detected, identified to sub-species level, and characterized. A significant benefit is detection of microorganisms in water that were previously missed or not identified by culture-dependent methods [22,26,27]. Metagenomic surveys carried out by other investigators have provided evidence that ingested diet-borne components can have short- and long-term effects on the human microbiota [28–31]. Only a few studies have used high-throughput sequencing to analyze DWDS, and the complete microbiome of finished drinking waters is vastly understudied.

This preliminary investigation is the first to use detailed and highly sensitive shotgun metagenomic high-throughput sequence analysis to identify components of microbial communities in order to describe the microbiome of DW. Metagenomic analysis of DW samples collected from a municipal tap, public drinking fountain, and BW, including sparkling natural mineral, spring, artesian, and reprocessed tap was employed. The relative abundance of bacteria, fungi, protists, bacteriophages, and virulence-associated factors was determined to provide an initial metagenomic survey of the total microbial content, including microorganisms in the viable but non-culturable state.

## Materials and methods

### Sample collection and preparation

DW samples collected in this study, including label, water type, source, collection date, production date, best-before date, storage container characteristics, major treatment steps prior to bottling, total and free residual chlorine concentrations, and volume of water analyzed, are described in **Table 1**. While the date and time of municipal tap (sample E) and drinking fountain (sample F) water samples leaving the water treatment plant (WTP) is not known, date and site of collection are provided. To analyze the DW microbiome and reduce the effect of premise plumbing, municipal tap and drinking fountain water samples (E and F) were collected after flushing the source water faucets. The municipal tap water faucet was flushed for 10 min and drinking fountain water faucet for 20 min prior to collecting 40 L of water in sterile Nalgene carboys (Thermo Fisher Scientific, Waltham, MA, USA) treated previously with hydrochloric acid (10% v/v), ethanol (95% v/v), and autoclaved. The drinking fountain water sample F was collected from a non-filtered, non-refrigerated, stainless steel Halsey Taylor OVL-II E Single Fountain (Halsey Taylor, Oak Brook, IL, USA). Tap and drinking fountain water samples were transported to the laboratory in a cooler box with ice and processed within one hour of collection to prevent growth, which would result in changes to the microbial community composition. Municipal tap and drinking fountain water samples were collected from the same location in Maryland, third floor of a building containing copper plumbing and approximately four miles from the municipal WTP supplying water to this location. The WTP employs free chlorine to disinfect water. Free chlorine concentrations in the water leaving the WTP during the sampling periods were reported by the WTP (5/9/2018 = 1.8 mg/L; 5/10/2018 = 1.9 mg/L; 5/11/2018 = mg/L; 6/21/2018 = 2.4 mg/L; 6/22/2018 = 2.5 mg/L; 6/23/2018 = 2.4 mg/L). Dates of purchase of sparkling natural mineral BW (sample A) and three non-mineral BW samples, including spring (sample B), artesian (sample C), and reprocessed tap (sample D) water types are also provided. Different brands of BW (samples A-D) were selected for study as our intent was to obtain a generalized knowledge of the DW microbiome. The brands selected did not disclose the exact source of their bottled waters. All BW samples were stored unrefrigerated until time of purchase. BW samples were stored at room temperature (23°C– 25°C) out of direct sunlight for up to one week after purchase since it was not possible to process all samples at the same time. BW brands in the interstate commerce are required to adhere to the standard of quality set by the USEPA, which requires a total residual chlorine concentration of less than 4 mg/L in finished DW [32]. Across BW samples selected for this study, the annual bottled water quality reports provided from each respective brand measured the total residual chlorine concentrations of water prior to bottling, and across all samples, the chlorine levels were below the minimum reporting limit set by the USFDA of 0.1 mg/L. Residual disinfectant was measured using a Pocket Colorimeter^TM^ II portable colorimeter (Hach, Loveland, CO, USA) in tap water from a building neighboring the sampling location through a shared distribution system on 5/7/2018 (total = 0.99 mg/L; free = 0.86 mg/L), 5/14/2018 (total = 0.91 mg/L; free = 0.69 mg/L), and 6/25/2018 (total = 0.78 mg/L; free = 0.65 mg/L). To represent the sampling event on May 11, 2018, the average of the total and free residual chlorine levels of May 7 and May 14 were taken (**Table 1**). As residual chlorine can cause complications during high-throughput sequencing, Safe Dchlor T20 sodium thiosulfate 20 mg tablets (Brim Technologies Inc., Randolph, NJ, USA) tablets were added to tap and drinking fountain water samples, per manufacturer’s specifications for dechlorination.

**Table 1:** Drinking water samples included in the study.

Sample	Water Type	Source	Collection Date (M/D/Y)	Production Date (M/D/Y)	Best-Before Date (M/D/Y)	Storage Container (Color/Material)	Major Treatment Steps Prior to Bottling	Total Residual Chlorine (mg/L)	Free Residual Chlorine (mg/L)	Volume Analyzed (L)
A	Bottled Sparkling Natural Mineral Water	Commercial	6/24/2018	3/15/2018	3/15/2021	Green/Glass	Injection of natural CO_2_			20
B	Bottled Spring Water	Commercial	5/11/2018	6/4/2018	12/31/2019	Clear/Plastic	Microfiltration; ultraviolet light and/or ozone disinfection			40
C	Bottled Artesian Water	Commercial	5/14/2018	1/15/2018	1/15/2020	Clear/Plastic	Microfiltration; ultraviolet light			9
D	Bottled Reprocessed Tap Water	Commercial	6/28/2018	5/30/2018	5/27/2019	Clear/Plastic	Reverse osmosis, ultraviolet light; ozone disinfection			40
E	Municipal Tap Water	Maryland, USA^a^	6/25/2018					0.78	0.65	40
F	Public Drinking Fountain Water	Maryland, USA^a^	5/16/2018					0.95^b^	0.775^b^	10

Respective sample label, water type, source, collection date, production date, best-before date, storage container characteristics, major treatment steps prior to bottling, total residual chlorine concentration, total free residual chlorine concentration, and volume of water concentrated are given.

^a^ Municipal tap and drinking fountain DW samples (E and F) were collected from the same location in Maryland.

^b^ Values represent an average of the total and free residual chlorine concentrations, respectively, collected on 5/7/2018 and 5/14/2018 from tap water near the sampling location.

All DW samples were concentrated by stepwise vacuum filtration at room temperature (23°C– 25°C) in sterile glass filtration units treated previously with hydrochloric acid (10% v/v), ethanol (95% v/v), and autoclaved. For each concentration, a total of 10 filters were used. Samples were passed through two 0.6 μm pore size polycarbonate Whatman Nuclepore Track-Etch Membranes (Millipore Sigma, St. Louis, MO, USA) which trapped trace minerals and expedited downstream filtration. The filtrate was aseptically collected and consecutively passed through two 0.2 μm and six 0.1 μm pore size polycarbonate Whatman Nuclepore Track-Etch Membranes (Millipore Sigma, St. Louis, MO, USA). However, because each water type contained a variable mineral content, the volume of water filtered was dependent on whether the filter clogged. Accordingly, the volume of water analyzed from each sample before the membrane filters clogged can be found in **Table 1**. The total filtrate passed through the two 0.6 μm filter membranes was subsequently processed as described. The 10 filter membranes for each sample were stored at -80°C until DNA preparation.

### Heterotrophic bacterial enumeration

Heterotrophic plate counts (HPC) of total bacteria were performed for BW (samples A-D) and, prior to dechlorination, tap water (sample E) and drinking fountain water (sample F), by direct and diluted (1/10 and 1/100) spread plating on BD Difco^TM^ R2A Agar (Fisher Scientific, Hampton, NH, USA), as previously described [33]. Incubation was at 24°C, and colonies were counted every 24 hours, for seven days to determine the HPC.

### DNA extraction and whole genome shotgun sequencing

Total DNA was isolated from the microbial biomass collected on all 10 filter membranes for each sample, using the ZymoBIOMICS^TM^ DNA Miniprep Kit (Zymo Research, Irvine, CA, USA), with the following modifications for DNA extraction from filter membranes. The 10 filter membranes for each sample were cut into ribbons approximately 2 mm by 10 mm and evenly distributed amongst five ZymoBIOMICS^TM^ Lysis Tubes, included in the ZymoBIOMICS^TM^ DNA Miniprep Kit (Zymo Research, Irvine, CA, USA). Final elution volume for each of the five preparations was 20 μl, and eluted DNA was pooled for each sample to 100 μl, respectively. DNA was purified using DNA Clean and Concentrator^TM^-25 Kit (Zymo Research, Irvine, CA, USA), following manufacturer’s instructions, with final elution volume of 50 μl.

Concentration of genomic dsDNA was measured using Qubit® dsDNA High Sensitivity Assay Kit (Thermo Fisher Scientific, Waltham, MA, USA) on an Invitrogen Qubit® 4.0 Fluorometer (Thermo Fisher Scientific, Waltham, MA, USA), which has a dsDNA quantification range of between 0.2 ng and 100 ng. Sparkling natural mineral BW and municipal tap and drinking fountain water samples yielded between 0.524–76.6 ng/μl of dsDNA (**Table 2**). However, dsDNA concentrations of spring, artesian, and reprocessed tap BW (samples B, C, and D) were below the limit of detection. To ensure sufficient genomic material was present in each sample that was required for subsequent library construction, 6.0 ng of *Pandoraea pnomenusa* KWW5 genomic DNA was added to samples B, C, and D. *P*. *pnomenusa*, serving as reference, is a Gram-negative bacterium of the family *Burkholderiaceae* and is frequently isolated from sputum of cystic fibrosis patients [34] and not expected to be present in finished DW in the USA. Genomic DNA used for spiking was prepared from pure cultures grown under standard conditions in BD Difco^TM^ LB Broth, Miller (Luria-Bertani broth; Fisher Scientific, Hampton, NH, USA), with aeration at 30°C overnight (16 hours) using the QIAamp DNA Mini Kit (Qiagen, Germantown, MD, USA), following manufacturer’s instructions.

**Table 2:** DNA Concentrations and sequencing statistics for samples included in the study as measured by FastQC.

Sample	Water Type	DNA Concentration (ng/μl)	Duplicate Reads (%)	Average GC Content (%)	Average Sequence Length (bp)	Total Sequences (Millions)
A	Bottled Sparkling Natural Mineral Water	0.524	38.0%	48.0%	186	10.5
B^a^	Bottled Spring Water	BDL	44.8%	63%	171	4.8
C^a^	Bottled Artesian Water	BDL	42.0%	63%	172	5.6
D^a^	Bottled Reprocessed Tap Water	BDL	54.2%	64%	187	30.0
E	Municipal Tap Water	76.6	27.4%	60%	146	19.9
F	Public Drinking Fountain Water	1.85	32.7%	54%	166	20.9
*P*. *pnomenusa* KWW5		68.0	38.7%	65%	153	6.7

^a^ Metagenomic reads contain sequence from *Pandoraea pnomenusa KWW5;* after removing spiked reads, 0.3, 0.1, and 0.4 million total sequences reads remained for samples B, C, and D, respectively.

BDL, below detection limit.

Genomic DNA libraries were constructed from the metagenomic samples and purified *P*. *pnomenusa* KWW5 genomic DNA, using the Thermo Fisher IonXpress Plus Fragment Library kit (Thermo Fisher Scientific, Waltham, MA, USA), following manufacturer’s instructions, with slight modifications for low-input DNA. Metagenomic and *P*. *pnomenusa* KWW5 DNA libraries were enriched and barcoded using the IonXpress Barcode Adapter Kit (Thermo Fisher Scientific, Waltham, MA, USA) and 13 cycles of PCR amplification, following manufacturer’s instructions. Resulting PCR products were purified using SPRIselect Reagent (Beckman Coulter, Indianapolis, IN, USA), following manufacturer’s user guide for next-generation library construction, and eluted in 25 μl low Tris-EDTA (TE) buffer (Thermo Fisher Scientific, Waltham, MA, USA). Final libraries were quantified by qPCR using the Ion Library TaqMan fQuantification Kit (Thermo Fisher Scientific, Waltham, MA, USA), which targets adapter sequences on each Ion Torrent library fragment. Sequencing was performed on an Ion S5 XL Semiconductor Sequencer (Ion Torrent, Thermo Fisher Scientific, Waltham, MA, USA) to generate 200 bp sequence reads, following manufacturer’s instructions. Operations and quality control associated with high-throughput sequencing, including a negative sequencing control, consisting of nuclease-free water, and a sequencing standard, i.e., ZymoBIOMICS^TM^ Microbial Community Standard (Zymo Research, Irvine, CA, USA), were done at CosmosID Inc. (CosmosID Inc., Rockville, MD, USA). Metagenomic samples were sequenced with an average of 1.5 x 10^7^ (min = 4.8 x 10^6^; max = 3.0 x 10^7^) sequence read depth across samples (**Table 2**). The low DNA input observed in BW samples B and C resulted in a slightly lower number of reads, 4.8 x 10^6^ and 5.6 x 10^6^, respectively, compared to the number of reads observed in samples A, D, E, and F and that employed in similar metagenomic investigations employing Ion Torrent chemistry [26].

### Metagenomic sequencing analyses

General sequencing statistics for all samples and mean sequence quality distribution, as measured by FastQC (v.0.11.6) [35], are detailed in **Table 2**. Base-calling error probabilities (P) were evaluated using Phred Quality Score (Q), defined by: *Q* = −10*log*_10_(*P*). Residual primer and adapter content were trimmed using the Joint Genome Institute Bestus Bioinformatics Decontamination Using Kmers (BBDuk) tool (v.38.07) [36] with a previously defined read quality trimming threshold [26]. Reads were trimmed from both ends until the mean quality value across each base position in the reads for all sample read libraries were above a Phred Quality Score of 17 for at least 80% of the read lengths, i.e., probability of correct base call was at least 98%. After quality trimming, the average Ion Torrent sequencing read lengths across libraries were between 146 bp and 186 bp.

To remove spiked *P*. *pnomenusa* KWW5 sequences from the metagenomic sample read libraries, the single *P*. *pnomenusa* KWW5 read library was assembled using the St. Petersburg genome assembler (SPAdes) software (v.3.12.0) [37] and options ‘—iontorrent’, required when assembling Ion Torrent data, ‘—s’, to specify a single read library, ‘—careful’, to reduce the number of misassemblies, and ‘—cov-cutoff auto’, to remove potentially mis-assembled low coverage contigs. The Translated Basic Local Alignment Search Tool (TBLASTX) was used to search the National Center for Biotechnology Information (NCBI; Bethesda, MD, USA) genome database using the largest contig (450,794 bp) from the KWW5 assembly as query sequence against *P*. *pnomenusa* published genomes. A subject database was built locally from the top five genome nucleotide sequences identified (GenBank Accession Numbers: CP015371.1, CP009553.3, CP006900.2, CP006938.2, CP007506.3) and the KWW5 draft assembly (94 contigs, scaffold sequence total = 5.504 x 10^6^ bp, L_50_ = 2.774 x 10^5^ bp). Raw metagenomic sample reads were mapped to the local *P*. *pnomenusa* database using the Burrows-Wheeler Aligner Maximal Exact Match (BWA-MEM) algorithm with default parameters from the Burrows-Wheeler Alignment Tool (v.0.7.17-r1188) [38]. Mapped reads were removed from the read libraries using SEQTK (v.1.3-r106) [39]. Successful removal of *Pandoraea pnomenusa KWW5* genomic sequences was confirmed by mapping the unmapped read datasets against the local *P*. *pneomenusa* database as previously mentioned. A further quality assurance was performed by manually inspecting the total list of detected organisms following subsequent metagenomic analysis for incidence of the genus *Pandoraea—*which was not detected.

Unassembled metagenomic sequencing reads, with *P*. *pnomenusa* sequences removed, were analyzed as previously described [26,40–42] using the CosmosID Metagenomics Cloud Application [43] to achieve microbial identification to species, subspecies, and/or strain level and quantification of microorganism relative abundance. Analogously, antibiotic and virulence-associated genes present in each sample were identified by querying unassembled sequence reads against GenBook®, a proprietary series of extensive databases curated by CosmosID Inc. (CosmosID Inc., Rockville, MD, USA). Briefly, the platform uses a data-mining k-mer algorithm to disambiguate sequencing reads into the discrete genomes or genes comprising the particular sequences. The GenBook® databases are composed of over 150,000 microbial genomes and gene sequences representing over 15,000 bacterial, 5,000 viral, 250 protozoan, and 1,500 fungal species, as well as over 5,500 antibiotic resistant and virulence-associated genes. All metagenomic analyses were performed using a filtered dataset with default parameters of the CosmosID Metagenomics Cloud Application [44].

Relative abundance of bacterial taxa in each sample was used for principal coordinate analysis (PCoA), employing Bray-Curtis distance measure [45]. Analysis of community virulome and virome was achieved by identifying virulence genes and viruses based on percent coverage as a function of gene-specific k-mer frequency in each sample. Sunburst visualizations and a heatmap of organism specific k-mer relative abundance (percentage) for each sample, were generated using Krona [46] and Morpheus [47], respectively. All datasets used to generate sunburst visualizations and heatmap were normalized by reducing the total list of detected microbial species less than 0.5% relative abundance in each sample to represent ‘other’ microorganisms. *Acidovorax* spp. NO-1 (GenBank Accession Number: HM357240.1) was detected in sample A and *Plasmodium falciparum* FCC-2/Hainan (GenBank Accession Number: ABGW00000000.1) was detected in sample E. Following quality control, *Acidovorax* spp. NO-1 was removed from the list of detected microorganisms in sample A, but *Plasmodium falciparum* FCC-2/Hainan was not removed from the list of detected microorganisms in sample E because the detected relative abundance was less than 0.5% and was included as ‘other’ microorganisms.

## Results

### Total bacterial culture count

HPC performed employing R2A medium yielded growth for artesian BW at a concentration of 1.92 x 10^4^ CFU/mL, after incubation for 96 hr at 24°C. The other BW samples did not yield growth, even after incubation for up to seven days at 24°C. The abundance of total heterotrophic bacteria for municipal tap water and drinking fountain water was 7.3 x 10^4^ CFU/mL and 7.8 x 10^3^ CFU/mL, respectively, after incubation for 72 hr at 24°C.

### Metagenomics of drinking water samples

A total of six DW samples were collected in this study including municipal tap water, water from a drinking fountain, sparkling natural mineral BW, and three non-mineral BW samples. Volumes of up to 40 L of water were analyzed and sources and descriptions of each sample are provided in **Table 1**. Shotgun metagenomic sequencing, using total DNA prepared from the six DW samples, generated approximately 9.84 x 10^7^ reads across the raw sequence libraries. The spiked *Pandoraea pnomenusa KWW5* genomic sequences were removed from the sequencing libraries of spring, artesian, and reprocessed tap BW samples, yielding 5.88 x 10^7^ high-quality metagenomic sequences, with number of sequencing reads between samples ranging from 1 x 10^5^ reads in artesian BW (sample C) to 2.09 x 10^7^ reads in drinking fountain water (sample F) (**Table 2**).

**Fig 1:**
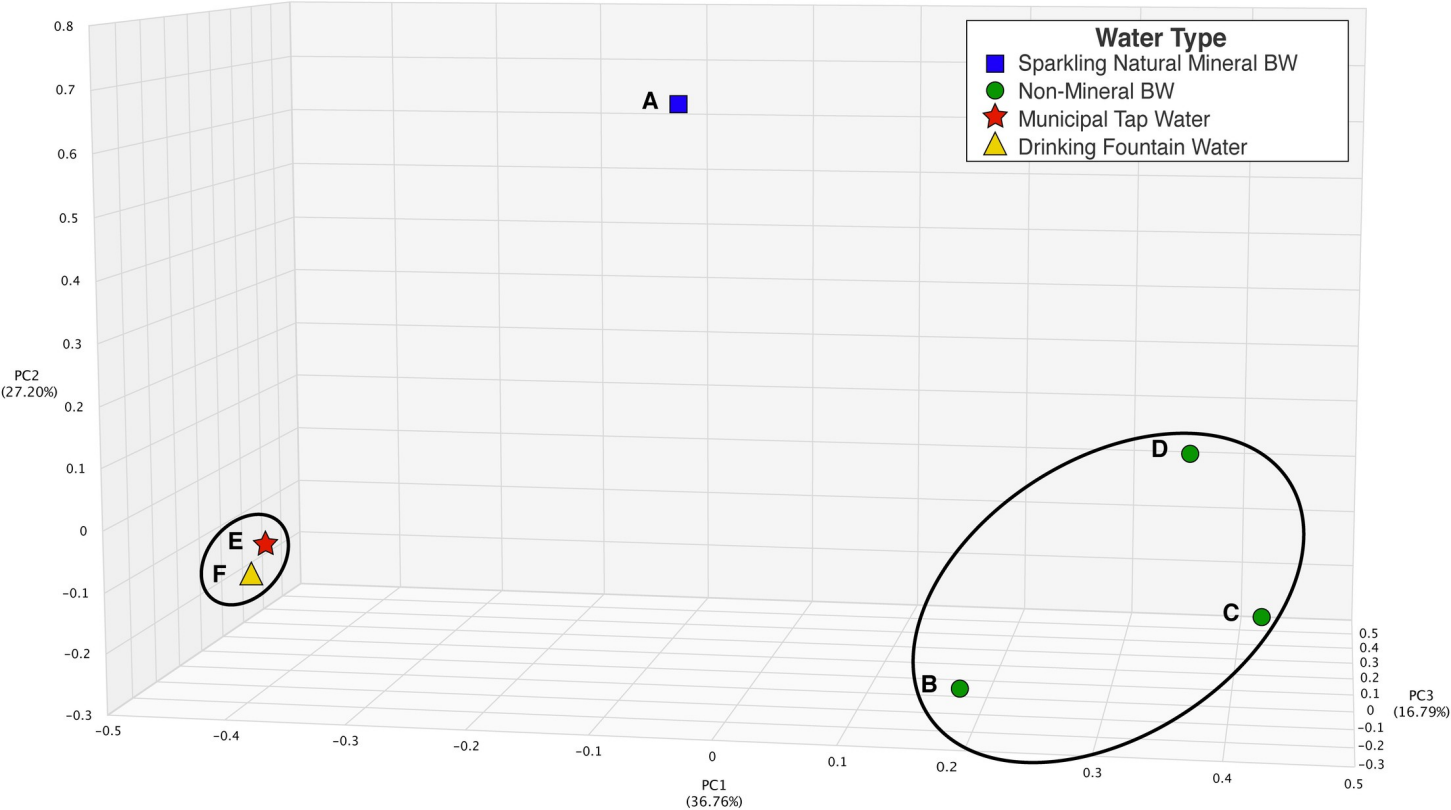
Principal coordinate analysis of bacterial communities in drinking water microbiomes.

Core bacterial communities of sparkling natural mineral BW, non-mineral BW (samples B, C and D), municipal tap water, and drinking fountain water were analyzed by three-dimensional Principal Coordinates Analysis (PCoA) using the Bray-Curtis dissimilarity index (**Fig 1**), where distance between points indicates degree of difference in bacterial DNA sequence composition. That is, points clustered more closely have similar microbiome composition. Each water type contained a relatively distinct bacterial composition across samples examined in this study. Non-mineral BW samples treated by microfiltration or reverse osmosis (samples B, C, and D) clustered together. Municipal tap water and drinking fountain water clustered more closely, compared to the other samples. Sparkling natural mineral BW (sample A) contained a bacterial composition unlike that of the other DW samples.

Relative abundance of bacterial species in each DW sample was analyzed by principal coordinate analysis using Bray-Curtis distance measure. Distance between points indicates degree of difference in bacterial DNA sequence composition, ranging from zero (samples share the same species abundances) to one (samples contain completely different species abundances). The percent of variation explained by each axis is indicated. Black circles are used to demonstrate distinct clustering observed across water types, i.e., municipal tap water clustered with drinking fountain water and the non-mineral bottled water samples clustered together, respectively. Blue square: sparkling natural mineral water, sample A; green circle: bottled non-mineral water, samples B, C, and D; red star: municipal tap water, sample E; yellow triangle: public drinking fountain water, sample F.

**Fig 2:**
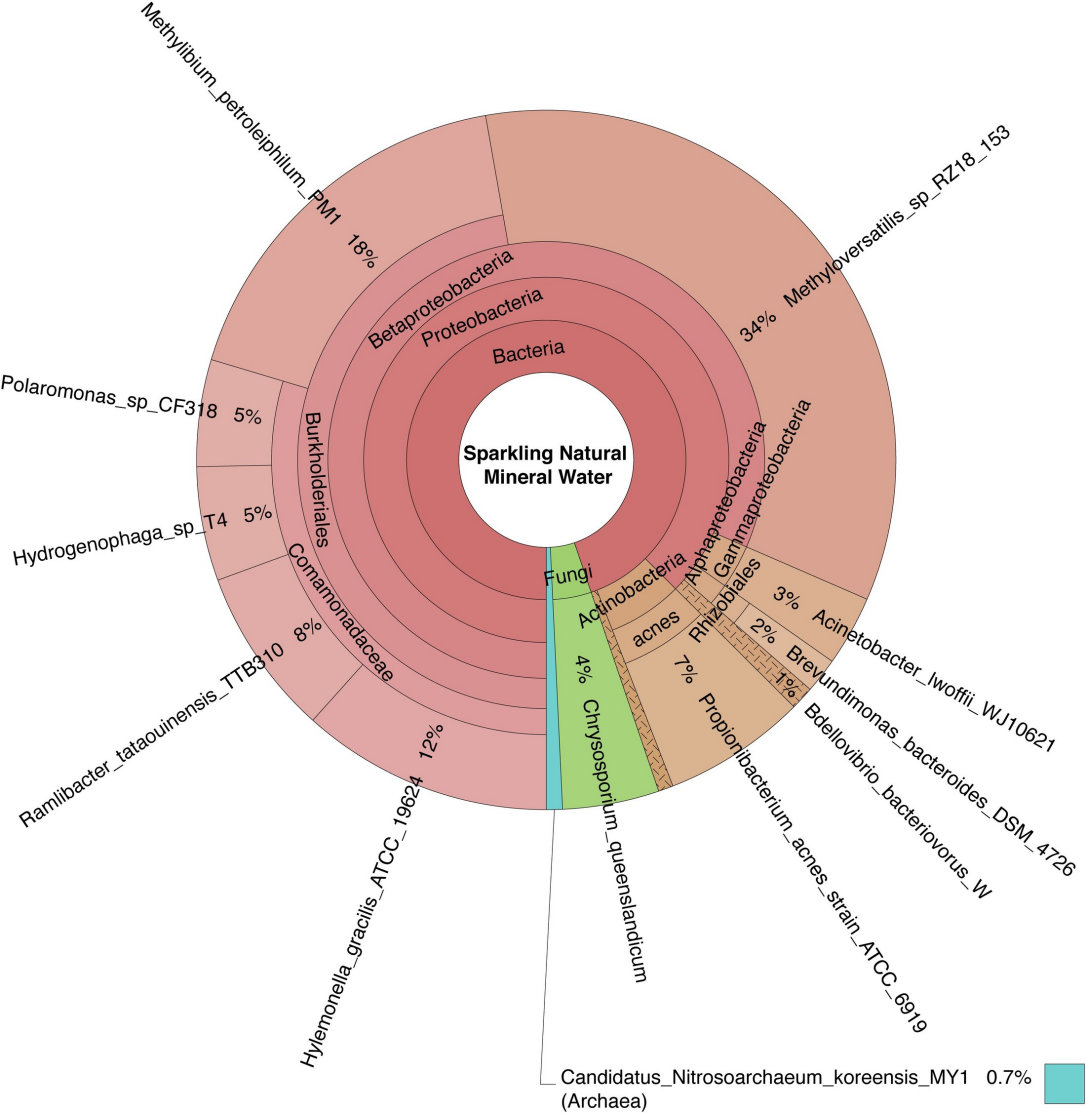
Krona plot of bottled sparkling natural mineral water microbiome. Species composition percentages are displayed as the normalized proportion of organism specific k-mers observed relative to the total microbial species diversity detected in the sample. Red, bacteria; green, fungi; purple, protozoa; teal, archaea.

**Fig 3:**
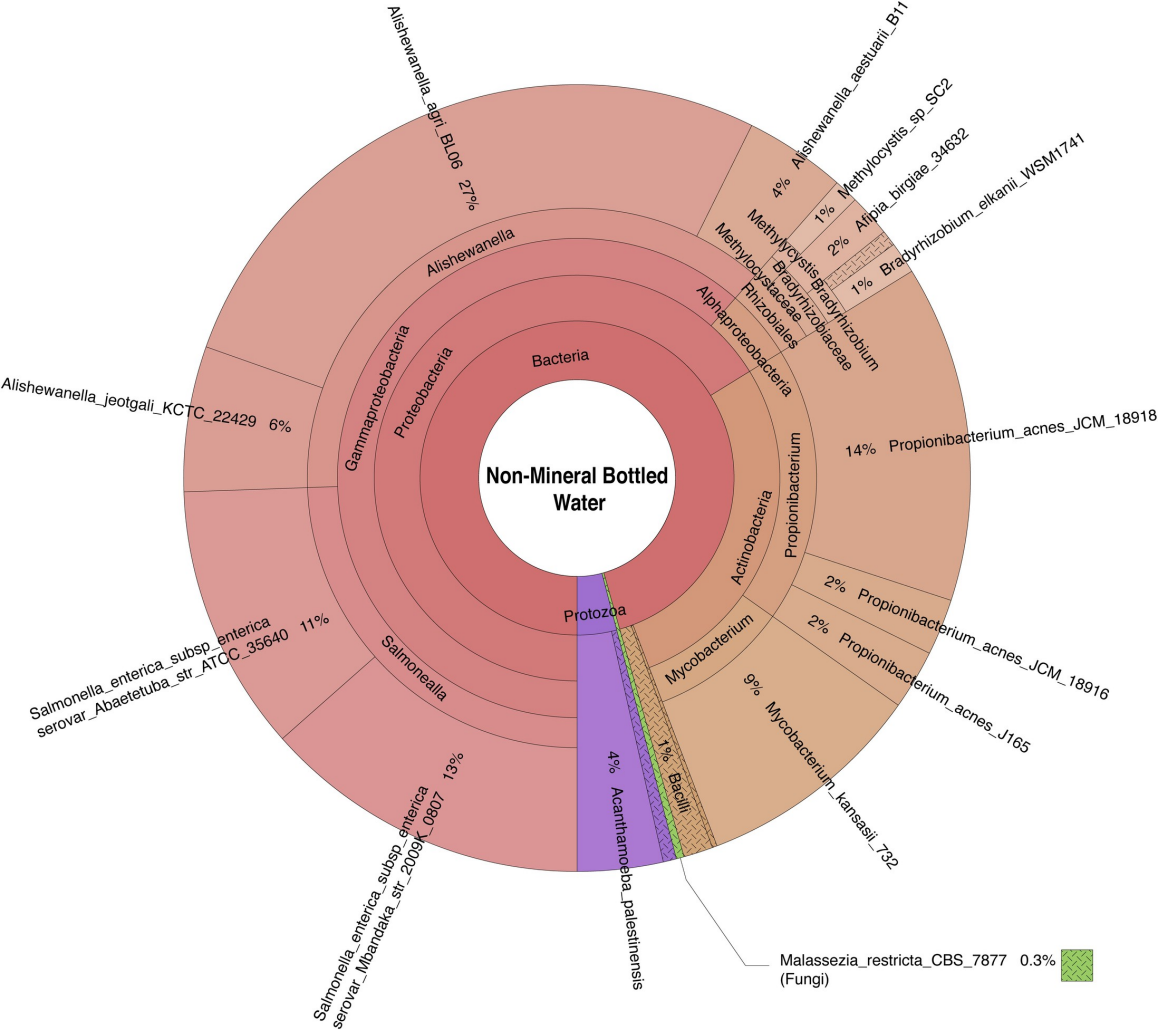
Krona plot of normalized bottled non-mineral water, including spring, artesian, and reprocessed tap water sample microbiomes. Species composition percentages are displayed as average number of organism specific k-mers detected, normalized to represent the proportion of organism specific k-mers observed relative to total microbial species diversity detected. Red, bacteria; green, fungi; purple, protozoa; teal, archaea.

**Fig 4:**
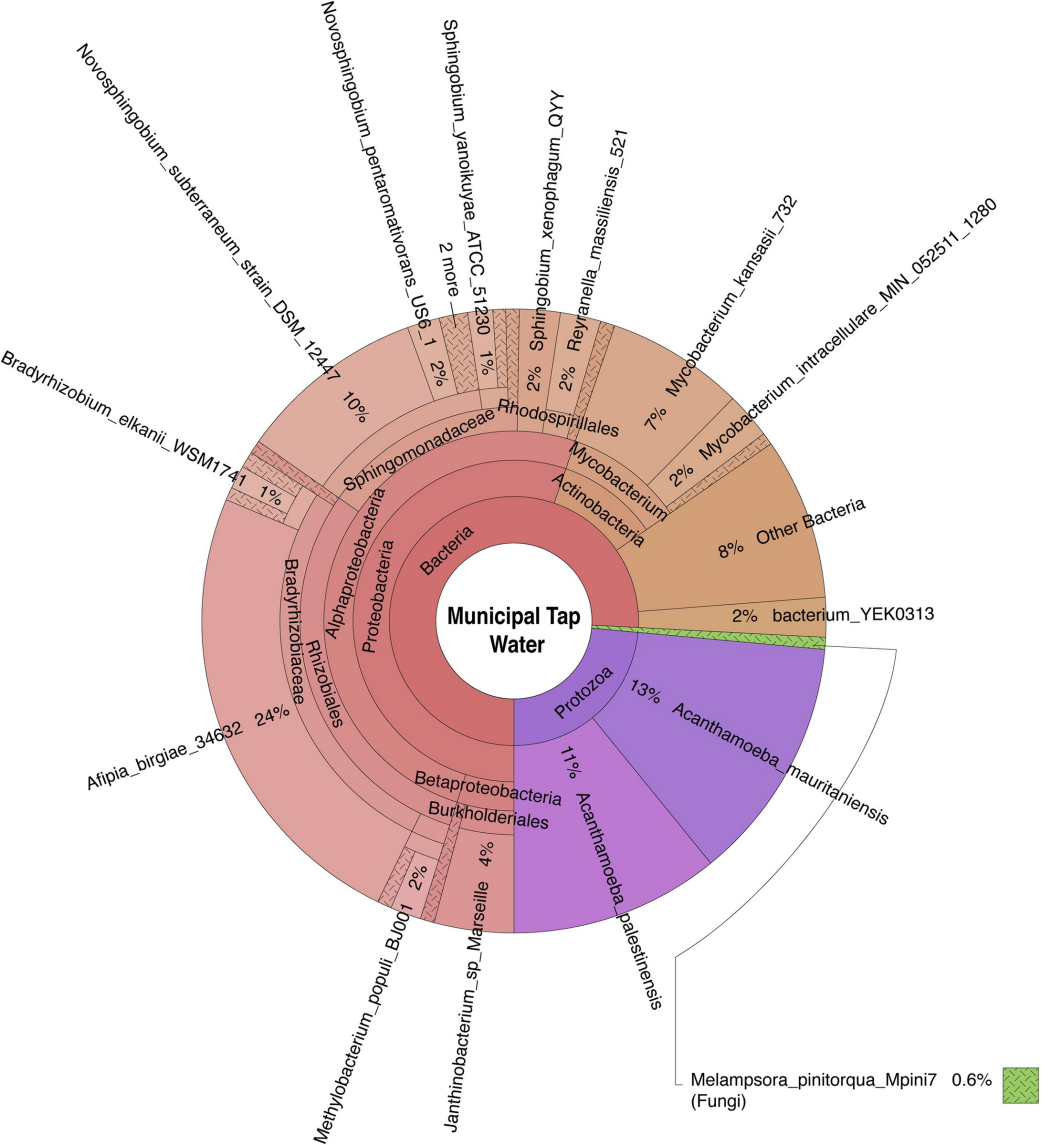
Krona plot of municipal tap water microbiome. Species composition percentages are displayed as the normalized proportion of organism specific k-mers observed relative to total microbial species diversity detected in the sample. Red, bacteria; green, fungi; purple, protozoa; teal, archaea.

**Fig 5:**
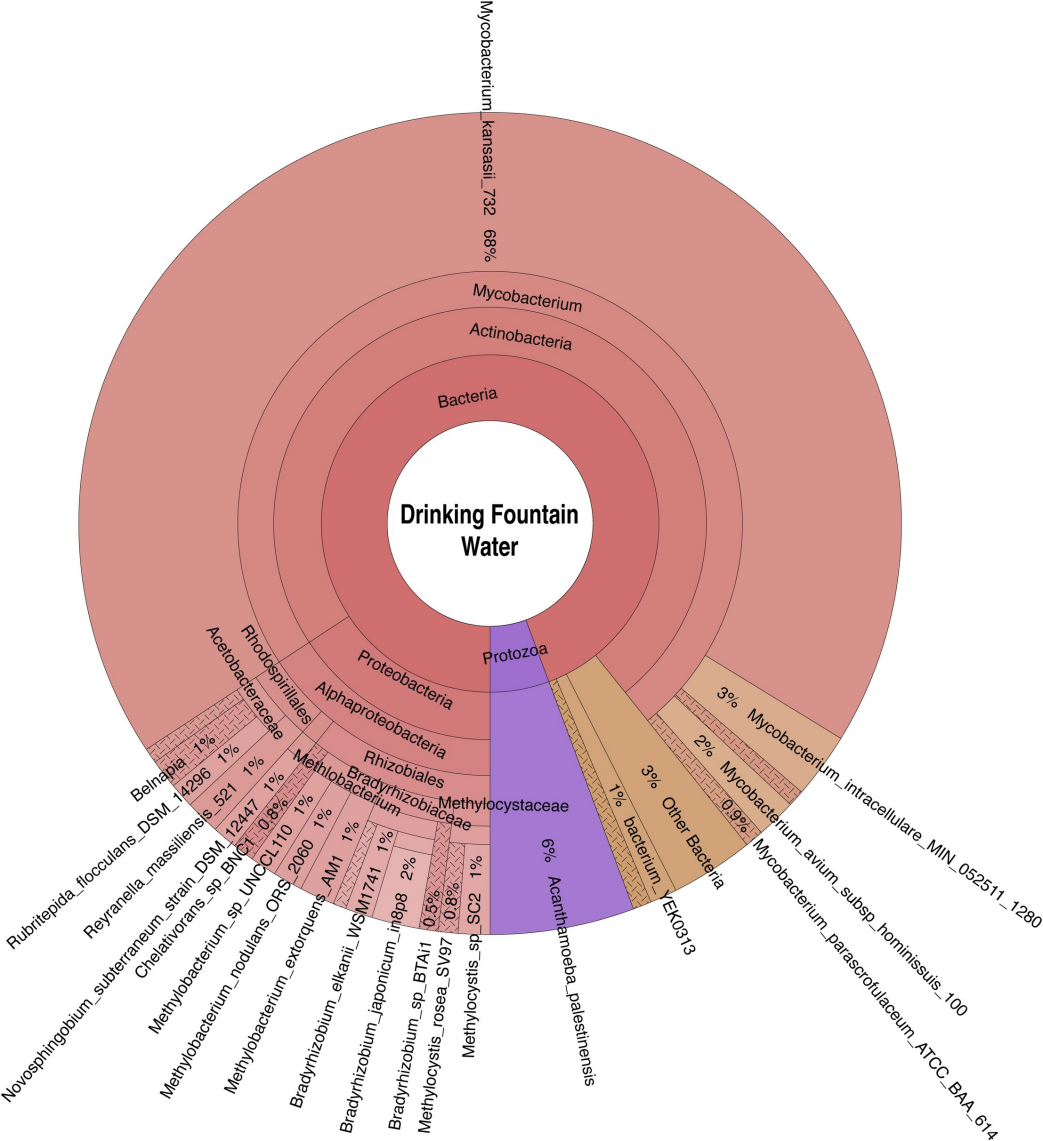
Krona plot of public drinking fountain water microbiome. Species composition percentages are displayed as the normalized proportion of organism specific k-mers observed relative to the total microbial species diversity detected in the sample. Red, bacteria; green, fungi; purple, protozoa; teal, archaea.

**Fig 6:**
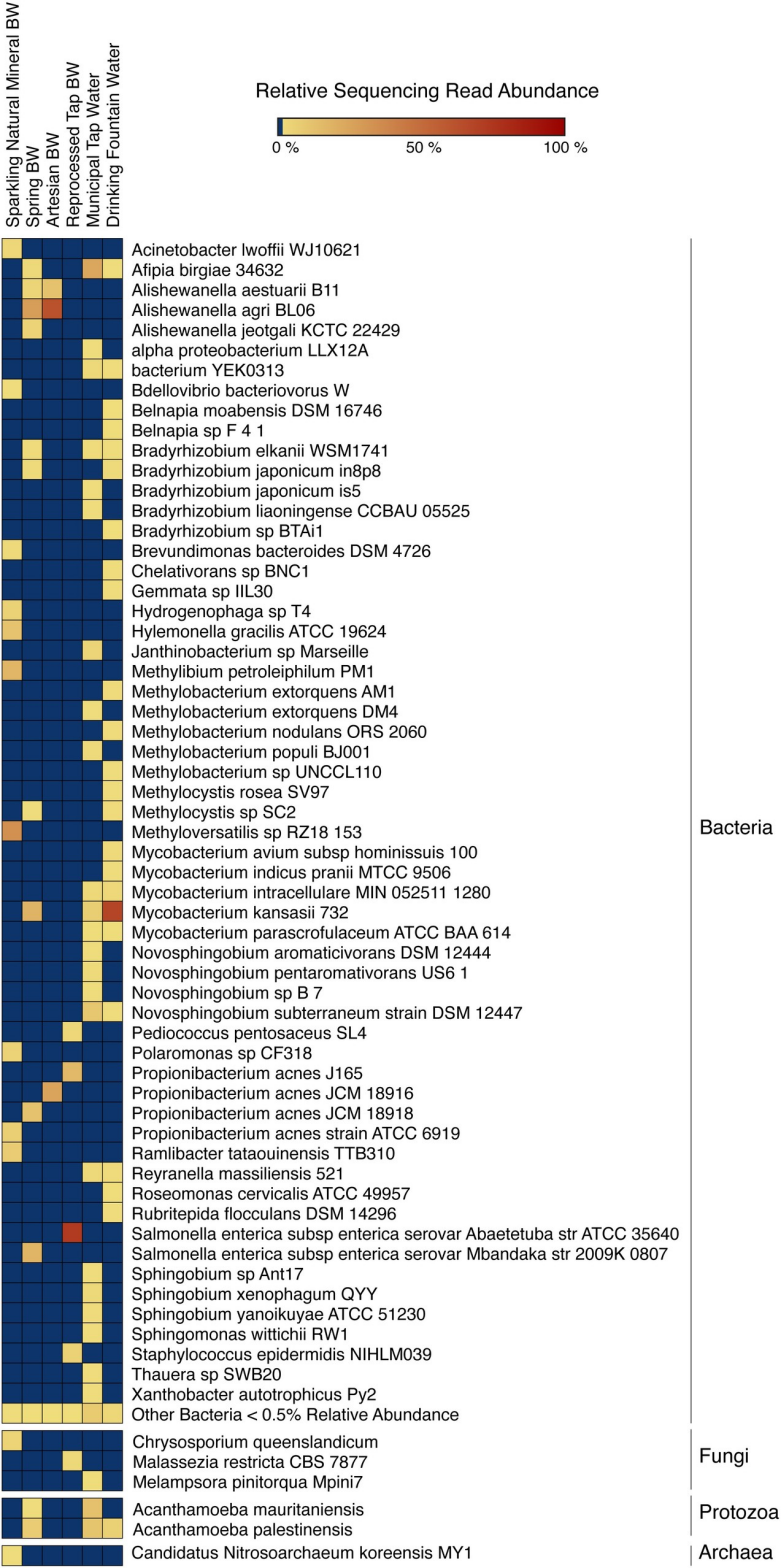
Heatmap of relative abundance of bacterial, fungal, protozoan, and archaeal species DNA in drinking water microbiomes. Species composition percentages are displayed as the normalized proportion of the microorganism specific k-mers observed in each sample relative to the total microbial species diversity of the sample. Color gradient key displays the scale of relative abundance percentages. Sample A, bottled sparkling natural mineral water; sample B, bottled spring water; sample C, bottled artesian water; sample D, bottled reprocessed tap water; sample E, municipal tap water; sample F, public drinking fountain water.

Bacteria, archaea, fungi, and protozoa identified by DNA characterization are shown in Krona plots, representing relative abundance of microbial species detected in sparkling natural mineral BW (Fig 2), non-mineral BW, showing gamma-diversity, i.e., total species diversity, among spring, artesian, and reprocessed tap water (Fig 3), municipal tap water (Fig 4), and public drinking fountain water (Fig 5). Interactive Krona plots used to generate Figs 2–5 are available in the Supporting Information (S1 File). The heatmap in Fig 6 depicts relative abundance of microbial species detected in all DW samples.

Dominant bacterial phyla detected include Gram-positive *Actinobacteria* and Gram-negative *Proteobacteria* in all samples. The majority of the *Alphaproteobacteria* was detected in municipal tap and drinking fountain water (**Fig 4** and **Fig 5**), *Betaproteobacteria* in sparkling natural mineral BW (**Fig 2**), and *Gammaproteobacteria* in other types of non-mineral BW (**Fig 3**). *Deltaproteobacteria* were not detected. *Rhizobiales* were common to both municipal tap and drinking fountain water (**Fig 4** and **Fig 5**). *Burkholderiales* were dominant in sparkling natural mineral BW (**Fig 2**) and *Alteromonadales* and *Enterobacteriales* in other types of non-mineral BW (**Fig 3**). *Propionibacterium* was detected in both sparkling natural mineral BW (**Fig 2**) and non-mineral BW (**Fig 3**).

*Afipia birgiae* and *Novosphingobium subterraneum* accounted for 24% and 13% of the relative sequencing read abundance, respectively, in municipal tap water. *Methylobacterium* spp. were detected in in both municipal tap and drinking fountain water, while *Sphingobium* spp. were unique to municipal tap water. *Mycobacterium* spp. were dominant in drinking fountain water, at 74% relative abundance (**Fig 5**), and also detected in spring BW and municipal tap water (**Fig 6**).

Opportunistic *Mycobacterium* spp. detected were primarily plant and animal pathogens, with *M*. *kansasii* the most abundant non-tuberculosis mycobacterium (NTM), detected at 68% of the total sequencing read abundance in drinking fountain water and lower abundance in municipal tap water and spring BW at 7% and 18%, respectively. *Mycobacterium intracellulare* was detected in municipal tap and drinking fountain water, and *Mycobacterium avium* and *Mycobacterium indicus pranii* in drinking fountain water, and *Mycobacterium parascrofulaceum*, an NTM and non-MAC (*Mycobacterium avium* Complex) organism, in both tap and drinking fountain water.

BW samples showed less species richness and diversity than tap and drinking fountain water samples, with fewer bacterial species detected. *Bradyrhizobium japonicum*, *Mycobacterium kansasii*, *and Afipia birgiae* were detected in spring BW and municipal and drinking fountain water. *Methylocystis* spp. were detected in spring BW and drinking fountain water (**Fig 6**). Different strains of *Propionibacterium acnes* were detected in each of the BW samples but not in sparkling natural mineral BW or municipal tap and drinking fountain water (**Fig 6**). *Alishewanella* spp. were most common in artesian BW but also detected in spring BW (**Fig 6**). *Salmonella enterica* subspp. *enterica* serovars Abaetetuba and Mbandaka and were detected in spring BW and reprocessed tap BW, respectively (**Fig 6**). As *Salmonella enterica* subspp. *enterica* are important opportunistic bacteria, further validation of these strain calls was performed by visualizing read coverage of *Salmonella enterica* subspp. *enterica* serovar Mbandaka str. 2009K-0807 (NCBI GenBank Accession Number: AMRS00000000.1) and *Salmonella enterica* subspp. *enterica* serovar Abaetetuba str. ATCC 35640 (NCBI Reference Sequence: NZ_CP007532.1) for samples B and D, respectively (**S1 Fig**).

Sparkling natural mineral water (sample A) appears to have a distinctive microbiome compared to other samples examined in this study (**Fig 6**). The dominant bacterial species identified were *Methyloversatilis RZ18 153* and *Methylobium petroleiphilum*, accounting for 36% and 19% of the relative microbial species diversity. Bacterial species of the family *Comamonadaceae* were detected in sparkling natural mineral BW. *Candidatus Nitrosoarchaeum koreensis* of the Archaeal TACK (*Thaumarchaeota*, *Aigarchaeota*, *Crenarchaeota* and *Korarchaeota*) superphylum was detected at less than 1% and not in any other DW samples.

Fungi and protists were detected but only at low relative abundance (**Fig 6**). Fungi detected include *Chrysosporium queenslandicum* in sparkling natural mineral BW, *Malassezia restricta* in reprocessed tap BW, and *Melampsora pinitorqua* in municipal tap water. *Acanthamoeba palestinensis* was detected in all DW samples except artesian and reprocessed tap BW. *Acanthamoeba mauritaniensis* was detected in spring BW and municipal tap water.

Genes associated with virulence were detected in some of the DW samples above the predefined metagenomic dataset filtering criteria (**Table 3**). Virulence-associated genes were not detected in artesian and reprocessed BW samples, and only at low abundance in other BW samples, including the genes *Proteus mirabilis tnpA* in spring BW and *Salmonella infantis tnpR* in sparkling natural mineral BW. Virulence coding genes were more common in municipal tap and drinking fountain water samples, e.g., *Klebsiella pneumoniae tnpA* and *Pseudomonas aeruginosa GI 3342496* and *Enterobacter aerogenes tniB*. Antibiotic resistance coding genes were not detected at a frequency to meet the predefined confidence levels set by the metagenomic analysis.

**Table 3:** Number of unique sequencing reads associated with bacterial virulence genes detected in the metagenomic analysis of drinking water DNA.

Sample	Water Type	Source Organism	Virulence Gene	Gene Function	Number of Unique Reads
A	Bottled Sparkling Natural Mineral Water	*Salmonella infantis*	*tnpR*	Resolvase	142
B	Bottled Spring Water	*Proteus mirabilis*	*tnpA*	Transposase	60
C	Bottled Artesian Water				0
D	Bottled Reprocessed Tap Water				0
E	Municipal Tap Water	*Klebsiella pneumoniae*	*tnpA*	Transposase	54958
		*Pseudomonas aeruginosa*	GI 3342496	Methyl-accepting chemotaxis-like protein	5913
		*Enterobacter aerogenes*	*tniB*	Transposase	264
F	Public Drinking Fountain Water	*Pseudomonas aeruginosa*	GI 3342496	Methyl-accepting chemotaxis-like protein	4003
		*Klebsiella pneumoniae*	*tnpA*	Transposase	463

Viruses were detected at a very low abundance and all were dsDNA bacteriophages (**Table 4**). These included *Salmonella* bacteriophages *vB_SemP_Emk* and *Fels-2* in spring BW and *Staphylococcus* bacteriophage *PvL108* and *Pseudomonas* bacteriophage *Pf1* in reprocessed tap BW and none in any of the other DW samples.

**Table 4:** Number of bacteriophage sequencing reads detected by metagenomic analysis drinking water DNA.

Sample	Water Type	Bacteriophages	Gene Function	Number of Unique Reads
A	Bottled Sparkling Natural Mineral Water			0
B	Bottled Spring Water	Salmonella phage vB_SemP-Emek	O-antigen modification	147
			[48,49]	
		Salmonella phage Fels-2	Cell lysis [50]	470
C	Bottled Artesian Water			0
D	Bottled Reprocessed Tap Water	Staphylococcus phage PvL108	Transposase [51]	18
		Pseudomonas phage Pf1	Filamentous bacteriophage [52]	34
E	Municipal Tap Water			0
F	Public Drinking Fountain Water			0

## Discussion

### Total viable bacterial counts

Currently, HPC is used to measure overall bacteriological quality of DW. Reprocessed tap BW in the U.S. interstate commerce is usually purified using a variety of steps, including conventional coagulation, flocculation, sedimentation, distillation, microfiltration, ozonation, reverse osmosis, and ultraviolet (UV) light treatment, to ensure the finished product meets USFDA standards derived from USEPA national DW standards—which does not intend for the final product to be sterile [53]. Having a high HPC in BW does not necessarily correlate with poor quality water, and heterotrophic regrowth in BW upon storage is common [18,54–56] due to the lack of a residual disinfectant being present [15]. Artesian BW, the only BW sample in this study to yield a positive HPC, did not contain added disinfectant residual but the sample had been collected post-treatment (**Table 1**), and the possibility of introduction during the bottling process cannot be ruled out. No residual disinfectants were present in the artesian BW sample included in this study, and the HPC(1.92 x 10^4^ CFU/ml) was similar to heterotrophic bacterial counts obtained in tap water collected from an intensive care unit (2.4 x 10^4^ CFU/ml) [22] and is within the HPC magnitude (10^4^ CFU/ml) observed in DWDS when the residual chlorine is less than 0.1 mg/L [57].

### Microbial diversity of drinking water

Bacterial phyla detected in DW (**Figs 2–5**) were similar to those commonly detected in municipal DWDS [22,58–61] and natural mineral BW [18,62], with *Proteobacteria* the most abundant. However, *Actinobacteria* was also detected in all DW samples examined.

A culture-independent study focused on the microbiota of DWDS, using 16S rRNA sequencing [22], identified *Alpha-* and *Beta-proteobacteria* subclasses as dominant bacterial communities of a water distribution network, but determined the Gammaproteobacteria subclass represented a relative abundance of less than 1%. Other studies of the microbiome of natural mineral water reported *Alpha*-, *Beta-*, and G*ammaproteobacteria* at moderate abundance [22,59,62]. In the study reported here, *Alphaproteobacteria* was dominant in municipal tap and drinking fountain water (**Figs 4 and 5**), *Betaproteobacteria* in sparkling natural mineral BW (**Fig 2**), and *Gammaproteobacteria* a relative majority in non-mineral BW (**Fig 3**).

Bacterial genera detected in municipal tap and drinking fountain water samples were similar to those frequently detected in DWDS [22,58–60]. *Mycobacterium* spp. were dominant in spring BW. *Mycobacteria* are commonly resistant to ozone- and chlorine-based disinfectants, two primary methods used to treat DW [63]. Biofilm production of *Mycobacteria* in DWDS has also been observed [10,61,64]. Some *Mycobacterium* spp. are opportunistic pathogens. *Mycobacterium* spp. are divided into two major categories: 1) causative agent of tuberculosis, including *M*. *tuberculosis*, *M*. *africanum*, and *M*. *canettii*, which are spread through the air and rarely detected in water and 2) NTM, including those responsible for MAC, the cause of many diseases in animals [65] and occasionally associated with pulmonary disease in humans, primarily *M*. *avium* and *M*. *intracellulare* [66]. Environmental NTM, i.e., *M*. *avium*, *M*. *kansasii*, and *M*. *xenopi*, are frequently isolated from DW and hospital water distribution systems, and resistance to chlorine, biofilm formation, and commensal relationships with amoeba has been recognized as a factor contributing to their persistence in DWDS [67]. Aerosols are the major route of dissemination of NTM, which is important because a number of NTM are spore forming—which may contribute to their persistence in the environment [68]. Hypersensitivity pneumonitis has been traced to the presence of NTM in shower heads [69]. In the USA, higher concentrations of NTM have been reported in DWDS disinfected with monochloramine than in DWDS disinfected by chlorination [70]. Haig and colleagues used a high-throughput approach to determine that greater water age, i.e., combined DWDS residence time and home plumbing stagnation time, is associated with a greater relative abundance of *M*. *avium*, and DW from locations closer to WTPs contain more diverse NTM spp. [71]. The WTP supplying water to the municipal tap and drinking fountain water samples included in this study use chlorine disinfection methods, yet presence of NTM was detectable. These findings point to the difficulty of eradicating NTM from premise plumbing, as consequence of their disinfectant-resistance and formation of biofilm [69], and highlight the importance of continued microbiological surveillance of DWDS.

Other genera detected in the municipal tap and drinking fountain water samples were *Afipia* and *Bradyrhizobium*, both common to the natural environments, specifically soil and water. However, it was recently demonstrated that the genus *Bradyrhizobium* is a common contaminant [72], including the 1000 Human Genomes Project [73]. It is possible that contamination of these bacteria was from the ultra-pure water used for DNA extraction and library preparation, since these organisms have an affinity for nitrogen flushed water [74].

*Alishewanella* was detected in spring and artesian BW samples. The genus *Alishewanella* belongs to the family *Alteromonadacease* and has been isolated from tidal flats [75], lakes [76], landfill soils [77], and fermented foods [78]. *Salmonella* was present in the reprocessed tap BW sample (**S1 Fig**) but at low abundance. Recognition of *Salmonella* outbreaks associated with fresh produce is relevant [79–81], and *Salmonella* spp. have been shown to survive and multiply in BW [82]. However, as can be seen from **Table 1**, the BW samples were collected post-treatment and considered finished DW. While *Salmonella* spp. were detected by DNA sequence analysis, growth on R2A was negative suggesting very low number of cells in the sample. Nonetheless, presence of *Salmonella* spp., particularly *S*. *enterica* subspp. *enterica* serovars Mbandaka and Abaetetuba, is important from a public health perspective as both serovars have been traced to culture confirmed *Salmonella* infections in the USA recently [83].

*Methylibium* spp. detected in sparkling natural mineral BW are hydrocarbon degrading organisms known to metabolize toluene, a solvent in many coatings used to protect municipal DW storage tanks [84]. *Methyloversatilis* spp. RZ18-153 was detected in sparkling natural mineral BW and is capable of utilizing single carbon (C_1_) compounds as sole source of energy [85]. *Methyloversatillis* spp. play an important role in H_2_/CO_2_-based membrane biofilm reactors that incorporate diffusions of H_2_ and CO_2_ to remove perchlorate [86] and may be naturally occurring or introduced via injection of natural CO_2_ into the sparkling natural mineral water.

*E*. *coli* and the *enterococcus* group, a subgroup of fecal *streptococci* including *Enterococcus faecium*, *Enterococcus durans*, *Enterococcus gallinarum*, and *Enterococcus avium*, are widely accepted as indicators of biological quality of DW [87,88]. Bacterial genera, e.g., *Enterobacter*, *Klebsiella*, *Citrobacter*, and *Escherichia*, have also been used as indicators of total coliform bacteria as they inhabit the intestinal tract of warm-blooded animals, but also soil, water, grain, and vegetation [89]. Fecal indicator and coliform bacteria have been reported in DWDS [90,91] and BW [92,93]. In the current study, no fecal indicator bacteria, i.e., *Escherichia coli* or *enterococci*, were detected in any of the DW samples analyzed. However, a transposase virulence factor coding for *Klebsiella pneumoniae*, a total coliform bacterium, was detected in municipal tap and drinking fountain water samples (**Table 3**). Transposases have potential to promote horizontal gene transfer across bacteria [94,95], and because *Klebsiella pneumoniae* was not detected in these samples, these genes might be indicative of horizontal gene transfer.

Prevalence of bacteria over archaea observed in this study is in agreement with previous reports [18,62,96]. *Candidatus nitrosoarchaeum* of the archaeal domain was detected only in sparkling natural mineral BW (**Fig 2**). *Candidatus nitrosarchaeum* is a very small rod-shaped archaea (diameter 0.3–0.5 μm and length 0.6–1.0 μm) that plays an important role in global nitrogen and carbon cycling [97]. This organism may occur more widely in DW than currently known, since other studies reporting on the microbiome of water employed 0.45 μm [82] or 0.2 μm [22,26] pore size filter membranes to concentrate the water samples before DNA extraction. These archaea would pass through those relatively large pore size filters. In this study, 0.1 μm pore size filter membranes were employed, making it possible to detect the *Candidatus nitrosarchaeum*.

Viruses and bacteriophages dominate the biosphere and have been reported to be present in some treated DW supplies [98]. Viruses are extremely host specific, and most phages can only infect a subset of bacterial species [99]. Some viruses, e.g., adenovirus, enterovirus, hepatitis A and E viruses, norovirus, and rotavirus, can cause a variety of human infections, including acute gastroenteritis [100]. In prokaryotes, the majority of viruses possess dsDNA genomes, while in eukaryotes, RNA viruses comprise the majority of the virome [101]. No known eukaryotic viruses were detected in any of the DW samples, and the bacteriophages that were detected were dsDNA viruses (**Table 3**). Specifically, the class II *Pseudomonas* phage Pf1, which can infect only those bacteria bearing retractile pili and not known to infect eukaryotes [52], was detected in reprocessed tap BW but in none of the other samples. Certain bacteriophages are important in overall microbial community structure and also major drivers of bacterial evolution [102]. Detection of *Salmonella* phages (vB_SemP_Emek and Fels-2) in spring BW provide confirmation of the presence of *Salmonella* spp.

Bacteriophages were readily detected in BW samples, but only below the limit of confidence in the municipal tap water sample and not in drinking fountain water (**Table 4**). Other studies have detected bacteriophages in municipal DW at high abundance. Méndez and colleagues found the concentration of bacteriophages outnumbered bacteria in metropolitan DW samples treated by chlorination while bacteria are detected more frequently than bacteriophages in springs, household water wells and rural water supplies [103]. A similar study done on chlorinated DW across three Canadian cities and determined the concentrations of bacteria were variable but all DW samples contained bacteriophages [104]. It is evident that bacteria and the phages that infect them respond differently to chlorination and other abiotic environmental influence in DWDS and finished DW. Additional microbiome investigation is needed to elucidate these complex interactions in DW.

Fungi can survive some water treatments and enter the DWDS post-treatment. Many fungal species survive in oligotrophic environments and are capable of growth by attaching to substrates that promote production of biofilm on pipe surfaces in DWDS [105,106]. Certain fungi, e.g., *Aspergillus* and *Candida*, pose serious health concerns for hospitals and health institutions, particularly immunocompromised patients [107,108]. Presence of fungi in DWDS and BW have been reported previously [109–111].

*Chrysosporium queenslandicum*, detected in the sparkling natural mineral BW sample belongs to the family *Onygenaceae*, and is not known to be a human pathogen. It has been used to hydrolyze keratinous debris and recycle poultry waste [112]. *Malassezia restricta*, found in the reprocessed tap BW sample is common to human skin and is a member of a group of yeasts detected in non-culture-based epidemiological studies [113]. *Melampsora pinitorqua* in the municipal tap water sample is a fungal parasite, known to induce pine twist rust in certain plant species [114].

*Acanthamoeba* spp. are ubiquitous free-living amoebae and function as predators, controlling microbial communities. These protists are common in the environment and previously have been detected in some domestic tap water samples [115], mineral BW, and laboratory distilled water [116]. In this study, *Acanthamoeba palestinensis* was detected in all DW samples except artesian and reprocessed tap BW. *Acanthamoeba mauritaniensis* was detected in spring BW and municipal tap water. *Acanthamoeba* spp. exist in two primary stages, one as a dormant cyst, and the other as an actively feeding and dividing trophozoite [116]. Under certain conditions, they have been recognized as opportunistic pathogens, which can be fatal or invalidating in humans and other animals, causing keratitis in immunocompetent individuals and cutaneous infection or granulomatous amoebic encephalitis in immunocompromised individuals [117,118]. Furthermore, *Acanthamoeba* spp., including *A*. *palestinensis* and *A*. *mauritaniensis*, have been shown to harbor opportunistic pathogens, particularly *Legionella* spp. [119,120], and are important to public health.

We observed prevalence of bacterial virulence genes to be higher in tap and drinking fountain water samples compared to BW samples (**Table 3**). It is possible that the low abundance of virulence factors detected in BW samples in this study can be attributed to a low amount of DNA for sequencing. A larger sample size and volume of water would allow for sequencing with higher coverage and yield a more complete characterization of microorganisms. Other studies employing molecular techniques have detected virulence factors in DW, including the DWDS [121,122], point of use tap water [123], artesian well water [124], mineral bottled water [124], and non-mineral bottled water [125].

Throughout this investigation, we were able to detect antibiotic resistance genes (ARG) at abundance levels below the limit of confidence. That is, ARGs were not present after implementing the confidence threshold. This finding differs from other molecular studies of DW where specific pathogenic bacteria harboring ARGs were detected in DWDS [126,127], bottled mineral water [128,129], and non-mineral BW [130]. Recent reports suggest that chlorination during treatment and distribution may enhance antibiotic resistance [13,14,131]. Stamps and colleagues demonstrated that during water treatment, microfiltration and reverse osmosis is effective in removing whole cells and transmissible genetic elements, including ARGs [121]. While the presence of antibiotic resistance genes in DWDS is important to public health, it is likely that the absence of antibiotic genes in DWDS is underreported. Collectively, these findings illustrate the need for microbiological monitoring of DW and DWDS to ensure water quality and safety.

Overall, this preliminary study reports increased bacterial species diversity in DW compared to previous findings where amplicon-sequencing and culture-dependent methods were employed. The whole genome metagenomic method employed in this study, despite total biological material recovered from BW being extremely low in concentration, provided a very rich set of useful information and new insight into DW microbiology warranting further assessments, relative to the public health significance of the non-traditional microbes present in various types of DW, and their relationships to the presence of indicator microorganisms.

## Limitations

With current status of whole DNA metagenomic sequencing technologies, investigators cannot conclude viability or infectious potential of the detected microorganisms. However, these approaches can utilize total DNA to detect accurately and identify all microorganisms in a sample, including bacteria, viruses, fungi, and protists, to sub-species level and characterize them. Primarily during concentration, there was variability in the volume of water analyzed. The dominant organisms detected in each sample would likely still be dominant if a larger sample volume was analyzed. During this investigation, a novel “DNA-spiking” approach was developed to increase the material used for DNA sequencing of samples with low input matrices. We chose to use purified genomic DNA of an unrelated organism, *Pandoraea pnomenusa* KWW5. Future metagenomic studies of samples with low biological content, e.g., water treated with reverse osmosis at water treatment facilities, may need to spike with human DNA or synthetic DNA of known DNA sequence that could be more effectively removed from the sequencing libraries. It is likely that the microbial communities detected in municipal tap and BW samples are water system, treatment, source, and possibly even seasonally specific. Further studies with water types would provide comprehensive analysis of the microbiome of DW.

## Conclusions

Whole DNA metagenomic sequencing and bioinformatics can be used effectively to study the autochthonous microbial community of DW and provide a powerful method for extracting new information on the quality of finished DW. Although they are valuable operational tools, the shortcomings of culture-based and indicator methods are well acknowledged, yielding only limited information on the microbiology of DWDS. This study provides an assessment of all microorganisms, bacteria, fungi, protists, and bacteriophages, present in various types of DW and allows an improved understanding of the microbial community structure of finished DW. This preliminary analysis, by applying whole genome metagenomics to determine the microbial composition of finished DW, has yielded new information on the microbial species composition of several drinking waters. Further investigation to address quantitative data will include additional samples and types of DW, and this work is in progress.

## Supporting information

S1 Fig Bottled water samples B and D sequencing coverage plots of *Salmonella enterica* subspp. *enterica*. (A) Bottled spring water (sample B) and (B) Bottled reprocessed tap water (sample D) sequencing reads were mapped against the genomes of *Salmonella enterica* subspp. *enterica* serovars Mbandaka (NCBI GenBank Accession Number: AMRS00000000.1) and Abaetetuba (NCBI Reference Sequence: NZ_CP007532.1), respectively, using the BWA-MEM algorithm with default parameters from the Burrows-Wheeler Alignment Tool [38].
(TIF)

S1 File Interactive Krona plots used to generate Figs 2–5. (HTML)
