# Supplementary figures and images for: A comparative analysis of drinking water employing metagenomics
Source: PLoS One. 2020 Apr 9;15(4):e0231210. doi: 10.1371/journal.pone.0231210 (PMC7145143; doi:10.1371/journal.pone.0231210)

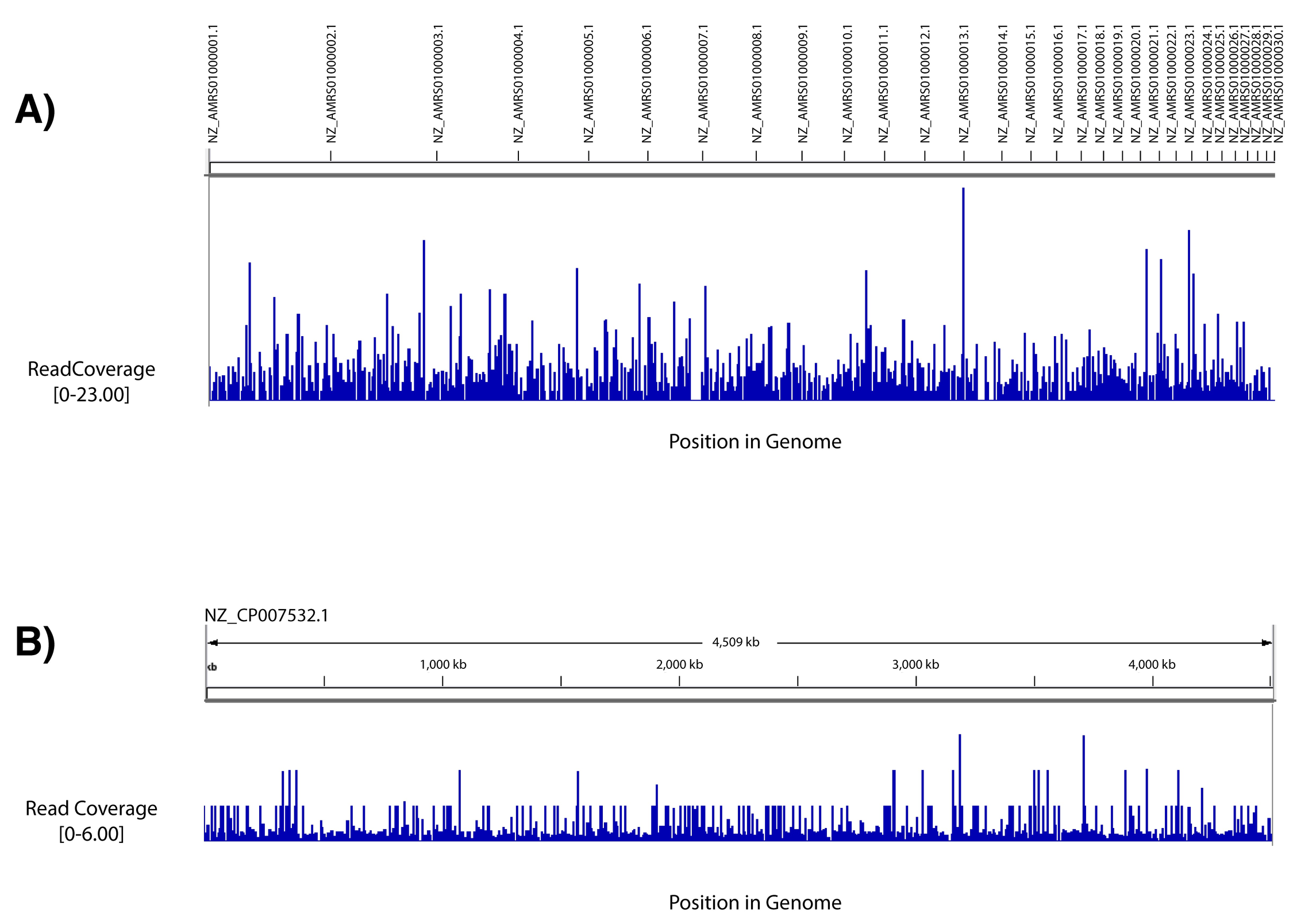

Supplement: S1 Fig — (A) Bottled spring water (sample B) and (B) Bottled reprocessed tap water (sample D) sequencing reads were mapped against the genomes of Salmonella enterica subspp. enterica serovars Mbandaka (NCBI GenBank Accession Number: AMRS00000000.1) and Abaetetuba (NCBI Reference Sequence: NZ_CP007532.1), respectively, using the BWA-MEM algorithm with default parameters from the Burrows-Wheeler Alignment Tool [38]. (TIF) [file pone.0231210.s001.tif]
